# Biomechanical Analysis of the Unaffected Limb While Using a Hands-Free Crutch

**DOI:** 10.3390/jfmk8020056

**Published:** 2023-05-04

**Authors:** Jaewook Kim, Yekwang Kim, Juhui Moon, Joo Kong, Seung-Jong Kim

**Affiliations:** Department of Biomedical Engineering, Korea University College of Medicine, Seoul 02841, Republic of Korea

**Keywords:** biomechanics, gait, kinematics, electromyography, hands-free crutch

## Abstract

Basic human ambulation relies on a bipedal gait, which has been reported to be directly related to quality of life. However, injuries to the lower limb can cause an inability to walk and require non-weightbearing periods to heal. Among the many ambulatory aids, standard axillary crutches are prescribed. However, due to the disadvantages of having to use both hands, a slow gait, pain, nerve damage, and gait patterns that differ from that of healthy subjects, currently, a new generation of ambulatory aids has emerged. Among such aids, hands-free crutches (HFCs) are of particular interest due to their form factor, which does not require the use of the hands and facilitates a bipedal gait. In this study, we present an assessment of whether any different gait patterns, compared to overground gait, appeared on the unaffected limb during walking with an HFC. The spatiotemporal parameters, plantar force, lower-limb joint angles, and EMG patterns were evaluated. In conclusion, the results from 10 healthy subjects suggest that wearing an HFC causes only slight changes in the biomechanical gait patterns examined in the unaffected limb compared with overground walking without an HFC.

## 1. Introduction

Bipedal gait is the first and foremost method of human ambulation and has been reported to be directly related to quality of life [1,2,3,4]. Gait can be readily performed by most people without much given effort, even though it requires complex control of individual muscles and whole-body balance [5,6,7,8,9].

However, injuries to the foot and ankle, which are frequently observed, can cause an inability to walk and require non-weightbearing periods in order to heal. In such cases, ambulatory aids are prescribed so that the non-weightbearing requirement is met while still providing some degree of mobility [10,11,12,13]. Among such aids, standard axillary crutches (SACs) provide a simple and economic means to suspend the affected limb so that the healing conditions can be optimized while the patient is still able to perform locomotion. It is estimated that over 575,000 people are prescribed with SACs each year for various reasons [10,14,15]. 

Unfortunately, SACs are not without shortcomings. Firstly, one or both hands are needed during ambulation; thus, in order to use one’s hands for other activities, it is most likely that one must come to a complete stop. Secondly, mobility is significantly reduced. Ambulation speed is decreased, even though there is a twofold increase in the energy demand compared with that of natural gait [16,17,18,19,20]. Thirdly, pain in the underarm, shoulder, ribs, and hands through the prolonged use of SACs can cause lasting nerve damage [12,21,22]. Fourthly, muscle atrophy of the affected limb may occur due to long-term disuse [23,24]. Lastly, the biomechanical patterns of the gait with SACs deviate significantly from the user’s natural gait [10,25,26,27]. 

**Figure 1:**
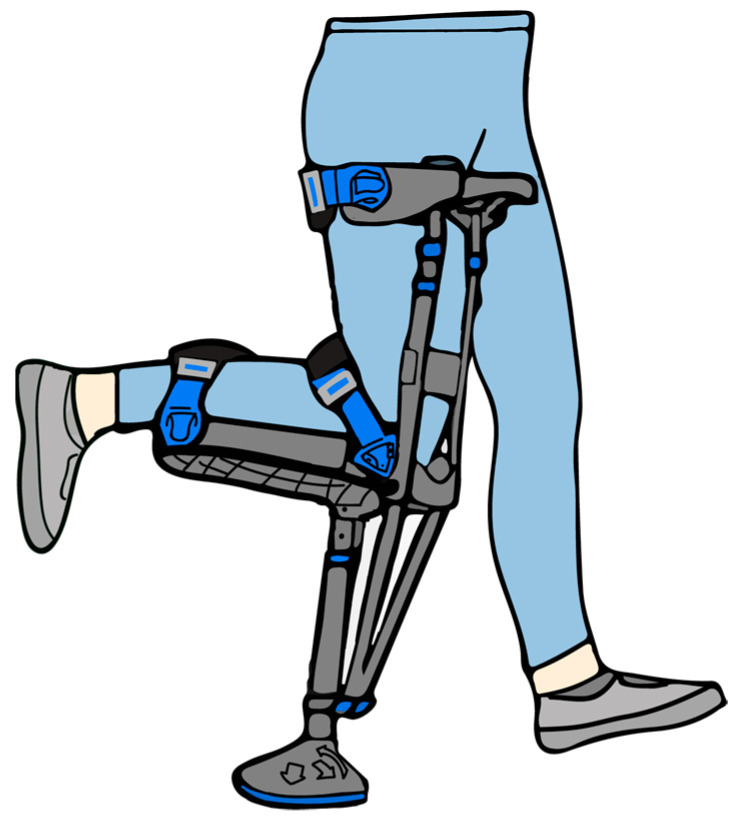
An illustration of the HFC used in this study (iWalk 3.0).

Recently, a movement towards a new generation of ambulatory aids that overcome the disadvantages of conventional SACs and promote a higher degree of freedom and better quality of life has emerged [10]. Among these interventions, hands-free crutches (HFCs) (Figure 1) are of particular interest because the form allows both hands to be free to engage in other activities, causes no pain in the upper limbs, and ensures no nerve damage [28,29]. Furthermore, previous studies by Dewar et al. have shown that ambulation with HFCs exhibits significantly higher levels of muscle recruitment within the affected lower limb, as shown by electromyography (EMG) signals, compared with what is observed when using SACs [24]. The significance of the results was that increased muscle recruitment could potentially reduce muscle atrophy [24].

While studies that have focused on the affected limb have been thoroughly conducted [24], there has yet to be an investigation, at least to our knowledge, of the biomechanics of the unaffected limb during ambulation with HFCs. The biomechanical patterns of the unaffected limb are important because adhering to compensation patterns for a prolonged period could cause detrimental habits [30]. 

Here, we present a pilot study which aims to evaluate whether any gait patterns that were different from overground gait without an HFC appeared in the unaffected limb during the gait with an HFC. The spatiotemporal parameters, plantar force, lower limb joint angles, and EMG patterns of movement with HFCs was compared with that of the natural gait at comfortable and slow speeds. In order to evaluate the data according to the gait-related tasks, the gait cycle was separated into four non-overlapping stages: double limb support 1 (DS1), single limb support (SS), double limb support 2 (DS2), and swing (SW). We hypothesized that the gait patterns of the unaffected limb during walking with an HFC would be similar to that of the natural gait and that not many compensation patterns would be introduced. The results from 10 healthy subjects suggest that, while slight changes were observed, the overall gait pattern of the lower limb remained intact.

## 2. Materials and Methods

### 2.1. Participants

Ten able-bodied subjects participated in this study. Volunteers with recent lower-limb injuries (within 6 months), severe medical conditions (or history of these conditions), and cognitive issues were excluded from this study. The participants were provided with a written informed consent form prior to participation; the research ethics of human experiments were ensured by conducting the study in accordance with the Declaration of Helsinki, and the study was approved by the Institutional Review Board of Korea University (IRB No. 2021-0120-01 on 1 April 2021).

### 2.2. Experimental Protocol

In order to assess the biomechanical effects of HFCs (Figure 1) during gait, the participants performed overground ambulation with and without an HFC (iWalk 3.0, iWALKFree Inc., Long Beach, CA, USA). While the participant walked over a pressure-sensitive 5 m walkway (GaitRite, SMS Technologies Ltd., Elizabeth Way, Harlow Essex, UK), the plantar force, joint angles, and EMG activation patterns were acquired via PedarX (Novel GmbH, Munich, Germany), Delsys IMU (Delsys Inc., Boston, MA, USA), and Delsys EMG sensors, respectively. The EMG data were obtained from the rectus femoris (RF), vastus medialis (VM), biceps femoris (BF), tibialis anterior (TA), gastrocnemius medialis (GM), and soleus (SL).

Data acquired from 3 overground gait conditions were compared: no HFC at a self-selected gait speed (baseline), no HFC at a slow speed, and with an HFC at a self-selected speed. In this experiment, all participants wore the HFC on the left limb. Because the right limb was the dominant limb for all 10 participants, we considered the right lower limb to be the unaffected limb throughout the study.

### 2.3. Signal Processing

The EMG data in this study were compared by calculating the waveform length (WL). The raw data were first modified with a bandpass filter (fourth-order Butterworth; 20 to 500 Hz). Then, the WL was obtained by Equation (1):

(1) $$EMG\;WL\left(n\right)=\overset{n}{\underset{i=n-N+2}{\sum }}\left|EMG\left(i\right)-EMG\left(i-1\right)\right|$$

where *n* is the current sample, *N* is the window size, *EMG* is the raw signal, and *EMG WL* is the EMG waveform length.

The difference in the EMG WL values from two different sensors cannot be directly compared [31]. Instead, normalized values must be prepared. In this study, the EMG WL signals were normalized using the difference from the baseline divided by the baseline, which was then represented as a percentage, as shown below:

(2) $$\Delta EMG\;WL_{Rel}=\frac{mean\;EMG\;WL_{condition}-mean\;EMG\;WL_{baseline}}{mean\;EMG\;WL_{baseline}}\times 100\%$$

The kinematic patterns of the lower limb were also evaluated. The joint angles of the hip, knee, and ankle were estimated on the sagittal plane. The 3D orientation data of the IMU sensors, provided via built-in sensors detecting acceleration, rotation, and the Earth’s magnetic field, were used to estimate the joint angles [32,33]. The IMU sensors were placed across the joint so that the relative orientation of the sensors represented the angle of the joint (refer to our previous study for details [34]).

**Figure 2:**
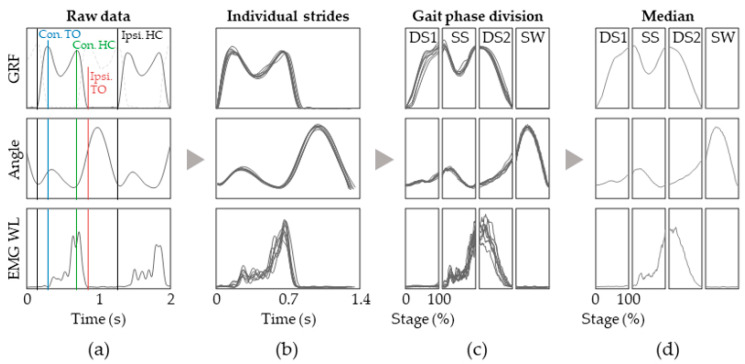
Signal processing and representation of data acquired during gait sessions. (**a**) The data are first synchronized and the ipsilateral heel contact (black lines) and toe-off (red lines) positions and contralateral toe-off (blue lines) and heel contact (red lines) are identified via applying a simple threshold on the GRF; (**b**) The data are then segmented into individual strides; (**c**) We divide the data into DS1, SS, DS2, and SW stage and then normalized into percentage of stage; (**d**) The median is plotted as lines.

In order to separate and group each stage, the EMG WL, lower limb joint angles, and plantar force data need to be synchronized on the time axis (Figure 2). Then, epochs such as the ipsi- and contralateral heel contact and toe-off positions were identified via GaitRite so that the synchronized data could be segmented into gait cycles (Figure 2b). The segments were further divided according to the 4 stages: DS1, SS, DS2, and SW. The first task, DS1, started from the ipsilateral heel contact and lasted until the toe-off of the contralateral limb. The SS task started immediately after the DS1 stage and ended with the heel contact of the affected limb. The DS2 stage was defined as from the end of SS to ipsilateral toe-off, followed by the SW stage, which concluded the gait cycle (Figure 2c). 

Plantar force sensitivity was also closely monitored in this study because it has been previously reported that it is closely associated with balance and the risk of falling [35,36]. Thus, it is imperative that foot pressure patterns different from walking without HFCs are not being acquired during HFC gait. The subject’s plantar force for each foot was acquired using Pedar-X insoles (50 Hz), which contain a matrix of 99 capacitive pressure sensors that span the entire insole region. We also used masks to obtain partial plantar force of the hind-, mid-, and forefoot to assess if any intra-foot changes in partial pressure were elicited. Furthermore, we evaluated the trajectory of the anterior–posterior center of pressure (CoP_ap_), as given by the Pedar-X insoles.

### 2.4. Group Analysis

It is vital to present common characteristics that are observed across the participant group and ensure traits that are only observed in a subset of the population are not presented as though they represented the entire group. Thus, each individual’s data were subjected to group analysis. Except for the kinematic data, a way to normalize and group the individuals’ data was needed. In this study, we used the $\Delta EMG\;WL_{Rel}$ for the EMG data, i.e., the *EMG WL* of a certain condition subtracted and divided by the baseline, represented as a percentage.

We also compared the shape of the EMG WL time traces by performing statistical analysis to determine whether they were similar or not. We determined $EMG\;WL_{norm}$, which normalized the EMG WL values such that the minimum value was 0 and the maximum value was 1, which was calculated as shown below:

(3) $$EMG\;WL_{norm}=\frac{EMG\;WL_{condition}-\min\left(\;EMG\;WL_{condition}\right)}{\max\left(\;\;EMG\;WL_{condition}-\min\left(\;EMG\;WL_{condition}\right)\right)}$$

The similarity between the two time traces of $EMG\;WL_{norm}$ was determined by evaluating the statistical significance, using Wilcoxon’s signed-rank test, of the median values of $EMG\;WL_{norm}$ for each gait stage.

### 2.5. Statistical Analysis

The plantar force data from each subject were divided by the individual’s bodyweight and represented as a percentage. The CoP_ap_ was normalized to the length of the insole so that it could be represented as percentage of the insole’s length. Once the data had been normalized, the median for each subject’s data was acquired, and the group data were presented as the median, and the 25th and 75th percentiles. We also evaluated the statistical significance by Wilcoxon’s signed-rank test. A custom MATLAB (Mathworks Inc, Natick, MA, USA) script was used for all the data processing.

## 3. Results

### 3.1. Temporal Parameters

**Figure 3:**
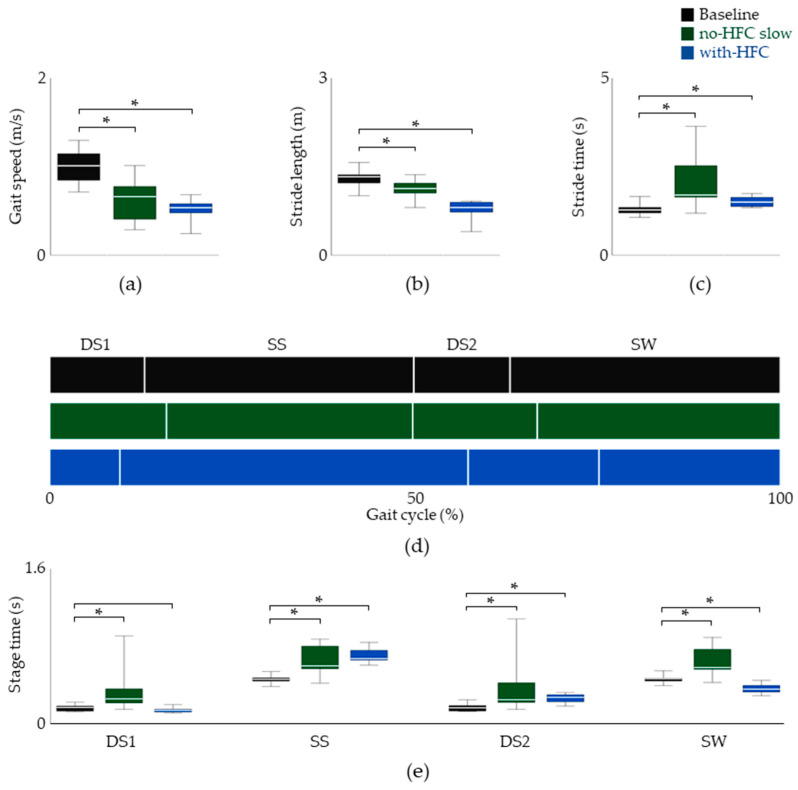
Spatiotemporal data of the unaffected limb. The gait speed, stride length, and stride time are shown in (**a**), (**b**), and (**c**), respectively. (**d**) The inter-stage ratio of stride time. (**e**) The duration of each stage. Data are shown as the median, and 25th and 75th percentiles. * *p* < 0.05, assessed using the Wilcoxon’s signed-rank test.

The temporal parameters were examined first. As seen in Figure 3, the speed and stride length for the natural gait at slow speeds decreased by 38.32% and 13.86% compared with the baseline, respectively. For the gait with the HFC, we found that the speed and stride length decreased by 46.15% and 39.31%, respectively (Figure 3a–c). However, an increase in the stride time was observed for both the slow gait and HFC gait. 

The stride time was further divided into the four gait stages so that the inter-stage ratios could be assessed (Figure 3). The data suggested little change during the DS1 stage for ambulation with the HFC. However, a 53.69% increase and a 26.54% decrease were observed for the SS and SW stages, respectively. At this point, it seemed that the inter-phase relationship was disrupted because of the longer and shorter duration of the ipsilateral and contralateral SS phases, respectively. 

### 3.2. Plantar Force and Kinematics

It is well-known that natural gait is energetically well-optimized in humans [5,37,38,39]. A major reason behind this is the transition between kinetic and potential energy [40,41]. Bipedal human gait transfers the potential energy to kinetic energy by utilizing heel, ankle, and forefoot rockers [42,43]. The kinetic energy after push-off is returned to potential energy, thus minimizing energy consumption. By analyzing the patterns of plantar force along with the kinematics of the lower limb, we assessed whether the natural patterns were preserved in the unaffected limb. Furthermore, because the foot of the unaffected limb is the only part of the body that directly reacts with the floor, the CoP trajectories were also carefully examined. 

**Figure 4:**
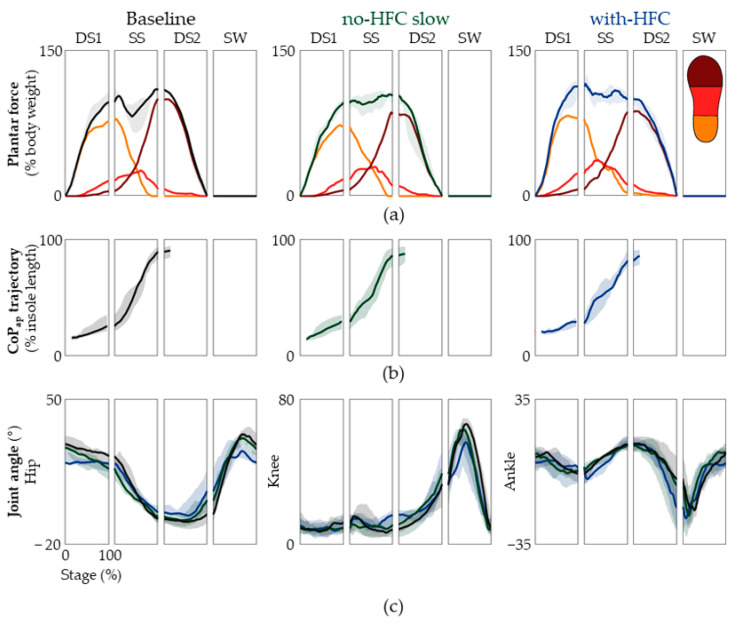
Plantar force patterns and kinematic data. (**a**) The overall plantar force of the hind-, mid-, and forefoot are shown. The baseline, slow speed with no HFC, and gait with an HFC are shown from left to right. The partial plantar force of the hind-, mid-, and forefoot is shown by yellow, orange, and dark red lines, respectively. The data are represented as the median and the 25th and 75th percentiles. (**b**) The trajectories of CoP_ap_ for the baseline, slow speed with no HFC, and with an HFC are shown from left to right. (**c**) The estimated joint angles of the hip, knee, and ankle. The data are represented as medians and the 25th and 75th percentiles.

**Figure 5:**
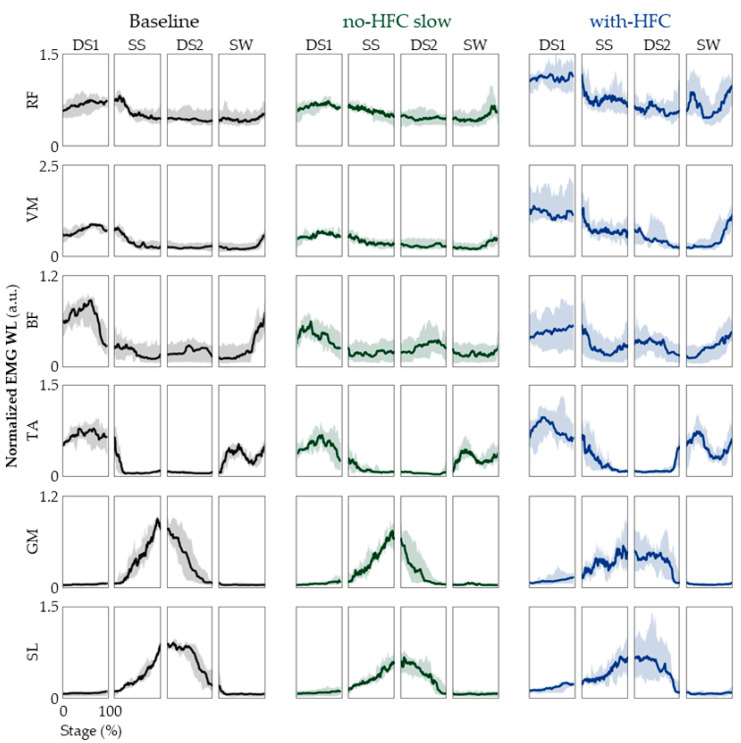
EMG patterns. The EMG WL time traces of the 4 stages are shown for RF, VM, BF, TA, GM, and SL. From left to right, the baseline (black lines), no-HFC slow (green lines), and with-HFC (blue lines) are shown. The data are represented as medians, and 25th and 75th percentiles.

The time traces of the overall plantar force and of the hind-, mid-, and forefoot are shown in Figure 4, with the median values presented in Table 1. For the full plantar force, we observed a 9.28% increase for the gait with an HFC in the SS phase. When we divided the plantar force into the hind-, mid-, and forefoot, there was no significant difference in the values, except for the hindfoot during the DS2 period; however, this value was only 0.57 % (Table 1). The results suggested that, in general, the global and regional plantar force patterns for the three conditions were quite similar. We next examined both the lower-limb joint angles and CoP_ap_ and assessed whether the rockers were well-facilitated. In order for a heel rocker to occur, the initial contact should be made in the heel region (Figure 4a,b), with the ankle dorsiflexed; plantar flexion should start after contact (Figure 4c, far right panel). The ankle rocker requires the entire foot to be in contact with the ground, and the ankle goes into dorsiflexion (Figure 4a,c). At this time, the dorsiflexor is not active (Figure 5); thus, this is passive dorsiflexion, meaning that dorsiflexion is elicited by the shin rolling over the foot. Finally, during forefoot rocking, the plantar force on the heel should be zero and the ankle should transition into plantar flexion (Figure 4c, far right panel). Furthermore, the median values shown in Table 1 suggested that there was no significant difference among the median CoP_ap_ values. However, we can see a decrease in the range of hip motion (Table 2). Collectively, we found that the unaffected limb during ambulation with an HFC facilitated the three distinct rocking motions, much like that of natural gait at both the self-selected and slow speeds but had a limited range of hip motion. 

### 3.3. EMG

One might now ask whether the user’s intent to recruit the muscles of the unaffected limb matches that of the natural gait. This question was evaluated by analyzing the EMG patterns of the lower limb, as shown in Figure 5 and Table 3. What is noteworthy is that we observed a marked increase in the EMG WL levels of the RF, VM, and TA for the gait with an HFC compared with the baseline. However, we were interested in the overall shape of the time traces, not the actual levels. Thus, we examined the values of $EMG\;WL_{norm}$, which are shown in Supplemental Figure S1 and Table 4. $EMG\;WL_{norm}$ transforms the time trace such that the values take on a value between 0 and 1. This allowed us to evaluate the shape of the time trace independent of the actual EMG WL levels (Supplemental Figure S1). While there were some differences within the different stages and muscles, overall, we did not see many significant differences (Table 4) [44,45].

## 4. Discussion

In this study, we presented an in-depth analysis of the lower limb’s EMG, joint angles, and plantar force patterns during gait with and without HFCs as a pilot study with 10 able-bodied participants. We noted that previous studies that have investigated gait with HFCs presented the EMG data as means over an entire gait cycle [24,46]. While conventional methods might be sufficient for assessing volitional intent [47,48,49,50], they fall short when depicting gait patterns, warranting a thorough comparison of the time traces. Thus, we qualitatively and statistically analyzed the data according to the distinct gait stages that comprise a gait cycle so that we could pinpoint where and how much the gait was affected by an HFC. With our approach, we found that the unaffected limb did not exhibit much deviation from the natural gait patterns.

We found that not only the total stride time but also the time for each stage differed significantly. What would cause such deviations? It is quite clear that the subjects were much more comfortable with a grounded unaffected limb and perceived the HFC to be more unstable. Thus, the time spent in the SW stage and the contralateral limb’s SS stage was shortened, which affected all the inter-stage ratios. This agreed well with the postulation that an unstable limb axis causes the swing phase to be shorter [51].

In order to assess the similarity in the time traces of the EMG WL, we needed to focus on the patterns and not the absolute values because the speed for each gait condition was different. It has been previously reported that the gait speed and recruitment of the soleus muscle are highly correlated [52]. Therefore, we examined the $EMG\;WL_{norm}$, which transformed the time trace so that the minimum value was 0 and the maximum value was 1. This allowed us to evaluate the shape independent of the absolute values. The results suggest all three gait conditions were mostly similar to one another. Specifically, as seen in Supplemental Figure S1, we observed the co-activation of the knee extensors and flexors at the beginning of the DS1 stage which provided stability for heel contact. The activation of these muscles decreased until the SW stage. We also observe dormant plantar flexors and activation of the dorsiflexors during the DS1 stage. As the EMG levels of the TA decreased, we observed an increase in the EMG levels of the GM and SL. The marked increase in the EMG level of the TA during the SW stage is vital for foot clearance and ensuring that the heel is the first part of the foot to contact the ground in the DS1 phase [44,45]. Taking into consideration that the joint angles of the lower limb and plantar force patterns were also similar, we were able to assert that natural gait is well-facilitated for the unaffected limb during the gait with an HFC without much compensation.

We noted that, while the patterns were similar, the EMG levels of the RF and VM in the DS1 stage were significantly higher even though the gait speed was reduced (Figure 4). This could be attributed to the HFC’s constant length, which cannot lower the center of mass during the contralateral SS stage. This increased the impact and required the extra recruitment of the ipsilateral RF and VM muscles during the DS1 stage.

Here, we divided the gait cycle into distinct stages, which was crucial for analyzing the gait patterns. By comparing the gait patterns of the unaffected limb during walking with HFCs with those of natural gait conditions, we were able to assess whether natural gait patterns were maintained for the unaffected limb. We found that the rocking motions of the heel, ankle, and forefoot were all intact, and the kinematic and EMG data and statistical analyses suggested that the overall gait patterns with an HFC were generally not significantly different from those of natural gait. While mostly conserved, we have some concerns about the contralateral SS stage (ipsilateral SW stage). We found a loss of hip extension and knee flexion during the SS stage. This could be attributed to the instability of the HFC, because the unafflicted swing phase is when the afflicted side is in the SS stage. We believe that it is necessary to perform a follow-up study of patients who have used HFCs for prolonged periods in order to check whether any different gait patterns were developed. Moreover, a future study on developing a spring-loaded device that would allow the user to slow down during the contralateral SS stage would be interesting. Lastly, once a patient has healed, it may be an option for them to wear the HFC on the unaffected limb as a training device in order to train the weakened affected muscles. In conclusion, the results from 10 healthy subjects suggest that wearing an HFC causes only slight changes in the biomechanical gait patterns examined in the unaffected limb compared with overground walking without an HFC.

## Figures and Tables

**Table 1:** The median plantar force and CoP_ap_ of the entire group.

Stage	Condition	Full (%)	Hindfoot (%)	Midfoot (%)	Forefoot (%)	CoP_ap_ (%)
DS1	Baseline	67.55 (63.31, 79.60)	62.96 (54.40, 79.14)	4.10 (2.38, 5.93)	0.56 (0.00, 1.31)	18.02 (16.60, 24.14)
	No HFC, slow	63.55 (55.73, 70.96)	53.23 (49.08, 59.23)	2.98 (0.88, 8.23)	1.10 (0.05, 1.78)	19.81 (15.53, 27.22)
	with HFC	81.65 (77.13, 85.87)	69.74 (57.68, 79.72)	6.30 (4.98, 11.54)	1.56 (0.89, 3.54)	23.86 (21.07, 26.65)
SS	Baseline	95.87 (83.30, 100.39)	28.90 (14.85, 38.50)	22.75 (16.80, 25.39)	31.72 (27.17, 39.42)	57.22 (44.33, 61.50)
	No HFC, slow	98.41 (89.22, 105.49)	31.45 (21.58, 43.23)	26.90 (24.70, 29.10)	32.15 (24.22, 39.62)	50.62 (41.34, 62.82)
	with HFC	104.78 * (95.43, 110.48)	33.00 (23.56, 44.33)	30.22 (24.06, 36.33)	34.91 (22.91, 47.39)	54.74 (45.29, 65.38)
DS2	Baseline	70.32 (62.91, 72.62)	0.00 (0.00, 0.00)	2.50 (0.77, 7.95)	65.05 (54.76, 69.42)	89.84 (83.60, 93.55)
	No HFC, slow	55.78 (35.96, 71.02)	0.00 (0.00, 0.00)	1.24 (0.00, 5.14)	51.44 (35.26, 66.38)	87.02 (77.85, 93.49)
	with HFC	72.21 (57.21, 83.46)	0.57 * (0.00, 2.24)	3.08 (0.54, 5.95)	66.00 (54.65, 74.27)	83.93 (77.33, 90.61)

Data are presented as the median (25%, 75%); * *p* < 0.05, assessed using Wilcoxon’s signed-rank test.

**Table 2:** The range of motion of the entire group.

Stage	Condition	Hip (°)	Knee (°)	Ankle (°)
DS1	Baseline	5.77 (5.15, 7.86)	8.80 (6.31, 9.96)	9.20 (7.77, 11.21)
	No HFC, slow	10.35 * (7.74, 14.08)	5.39 * (4.17, 6.74)	11.21 (9.73, 15.17)
	with HFC	4.07 * (3.29, 5.08)	7.42 (5.89, 7.96)	9.40 (6.12, 10.83)
SS	Baseline	31.78 (25.35, 34.47)	9.85 (5.81, 12.31)	16.58 (15.83, 18.25)
	No HFC, slow	23.80 * (18.20, 27.03)	6.37 (5.02, 10.04)	13.54 (8.98, 18.95)
	with HFC	23.64 * (21.66, 24.86)	7.75 (5.50, 10.31)	15.07 (11.59, 22.58)
DS2	Baseline	6.60 (3.01, 10.17)	25.31 (13.67, 28.03)	13.01 (9.54, 26.93)
	No HFC, slow	9.45 (6.47, 12.53)	26.96 (21.78, 30.28)	21.97 (16.79, 38.24)
	with HFC	12.14 (8.51, 17.13)	21.25 (13.85, 30.84)	31.48 * (23.61, 32.71)
SW	Baseline	40.31 (30.25, 44.45)	56.33 (50.41, 61.50)	31.02 (24.79, 39.83)
	No HFC, slow	33.02 (25.53, 36.92)	53.97 (51.87, 57.14)	29.62 (23.22, 40.67)
	with HFC	19.34 * (14.30, 28.18)	41.68 * (34.67, 55.01)	29.22 (23.00, 31.36)

Data are presented as the median (25%, 75%); * *p* < 0.05, assessed using Wilcoxon’s signed-rank test.

**Table 3:** The median ∆*EMG WL_rel_* of the entire group.

	DS1 (%)		SS (%)		DS2 (%)		SW (%)	
	No HFC, Slow	with HFC	No HFC, Slow	with HFC	No HFC, Slow	with HFC	No HFC, Slow	with HFC
RF	−3.57 (−11.04, 10.38)	76.22 * (41.39, 112.1)	0.16 (−5.37, 6.07)	47.84 * (15.51, 70.38)	2.08 (−9.55, 7.4)	33.32 * (14.6, 54.66)	3.19 (−7.91, 21.03)	66.57 * (17.66, 112.2)
VM	−14.84 (−22.7, 15.34)	98.85 * (43.22, 178.53)	−0.15 (−18.84, 32.59)	87.99 * (20, 157.19)	13.7 (3.1, 58.74)	93.35 (52.38, 114.13)	5.98 (−3.36, 28.44)	49.02 (22.75, 139.54)
BF	−32.08 * (−49.4, −22.39)	−11.38 (−49.64, 8.06)	−7.74 (−25.4, 13.15)	23.45 (8.31, 51.35)	20.3 (9.85, 43.61)	29.55 (1.93, 112.34)	−19.99 (−51.77, −5.77)	0.23 (−45.57, 18.58)
TA	−18.08 (−32.69, −1.67)	23.84 (−10.61, 61.78)	29.19 (−6.05, 49.45)	147.7 * (62.08, 193.44)	−9.06 (−16.19, 21.33)	76.37 * (35.02, 127.76)	−8.41 (−22.79, 12.65)	54.62 * (17.48, 112.29)
GM	7.78 (1.52, 83.69)	116.7 * (30.76, 234.81)	0.08 (−26.9, 14.34)	−5.84 (−37.53, 8.23)	−33.81 (−55.31, −17.45)	27.95 (−56.73, 53.77)	0.67 (−29.49, 7.67)	6.56 (−23.05, 13.71)
SL	6.34 (−1.71, 29.98)	100.46 (64.59, 115)	−12.92 (−26.34, −4.35)	10.98 (−14.7, 38.69)	−28.1 * (−48.92, −26.36)	−21.12 (−43.49, 53.99)	−7.15 (−16.41, 3.18)	15.7 (7.62, 30.85)

Data are presented as the median (25%, 75%); * *p* < 0.05, assessed using Wilcoxon’s signed-rank test.

**Table 4:** The ∆*EMG WL_norm_* of the entire group.

Stage	Condition	RF (%)	VM (%)	BF (%)	TA (%)	GM (%)	SL (%)
DS1	Baseline	0.50 (0.43, 0.62)	0.54 (0.45, 0.66)	0.82 (0.57, 0.91)	0.64 (0.57, 0.77)	0.02 (0.01, 0.04)	0.03 (0.02, 0.09)
	No HFC, slow	0.68 (0.46, 0.76)	0.70 (0.57, 0.86)	0.86 (0.57, 0.97)	0.82 (0.63, 0.97)	0.05 (0.02, 0.26)	0.10 * (0.05, 0.21)
	with HFC	0.38 * (0.19, 0.46)	0.48 (0.40, 0.54)	0.55 * (0.48, 0.60)	0.57 (0.40, 0.65)	0.27 * (0.05, 0.40)	0.10 * (0.07, 0.25)
SS	Baseline	0.88 (0.70, 0.97)	0.94 (0.77, 0.97)	0.52 (0.23, 0.74)	0.84 (0.47, 0.99)	0.98 (0.92, 0.99)	0.84 (0.78, 0.90)
	No HFC, slow	0.8 (0.62, 0.90)	0.83 (0.69, 0.95)	0.54 (0.28, 0.83)	0.77 (0.55, 0.93)	0.94 (0.78, 0.98)	0.84 (0.75, 0.90)
	with HFC	0.7 (0.46, 0.88)	0.81 (0.75, 0.89)	0.87 (0.50, 0.97)	0.74 (0.59, 0.99)	0.91 * (0.52, 0.97)	0.61 * (0.40, 0.67)
DS2	Baseline	0.29 (0.11, 0.41)	0.14 (0.08, 0.34)	0.15 (0.06, 0.37)	0.08 (0.06, 0.13)	0.76 (0.65, 0.93)	0.88 (0.58, 0.92)
	No HFC, slow	0.28 (0.18, 0.45)	0.39 (0.20, 0.46)	0.87 * (0.45, 0.98)	0.13 (0.08, 0.47)	0.79 (0.54, 0.91)	0.87 (0.77, 0.98)
	with HFC	0.36 (0.25, 0.62)	0.38 * (0.28, 0.55)	0.47 (0.27, 0.67)	0.56 * (0.09, 0.76)	0.79 (0.66, 0.90)	0.90 (0.61, 0.99)
SW	Baseline	0.69 (0.21, 1.00)	0.76 (0.34, 1.00)	1.00 (0.99, 1.00)	0.86 (0.67, 1.00)	0.05 (0.01, 0.40)	0.15 (0.05, 0.42)
	No HFC, slow	0.93 (0.33, 1.00)	0.78 (0.47, 1.00)	0.76 (0.43, 0.86)	0.87 (0.79, 1.00)	0.02 (0.01, 0.08)	0.08 (0.02, 0.10)
	with HFC	0.74 (0.45, 1.00)	0.66 (0.56, 1.00)	0.88 (0.74, 1.00)	0.74 (0.64, 0.87)	0.15 (0.08, 0.17)	0.10 (0.05, 0.24)

Data are presented as the median (25%, 75%); * *p* < 0.05, assessed using Wilcoxon’s signed-rank test.

## Data Availability

The data presented in this study are available upon request from the corresponding author.

## Supplementary Materials

The following supporting information can be downloaded at: https://www.mdpi.com/article/10.3390/jfmk8020056/s1.
