# Supplementary material for: Biomechanical Analysis of the Unaffected Limb While Using a Hands-Free Crutch
Source: J Funct Morphol Kinesiol. 2023 May 4;8(2):56. doi: 10.3390/jfmk8020056 (PMC10204483; doi:10.3390/jfmk8020056)
Supplement: Supplementary file 1 [file jfmk-08-00056-s001.zip › jfmk-2248013-supplementary.pdf]

Journal of
Functional Morphology and Kinesiology

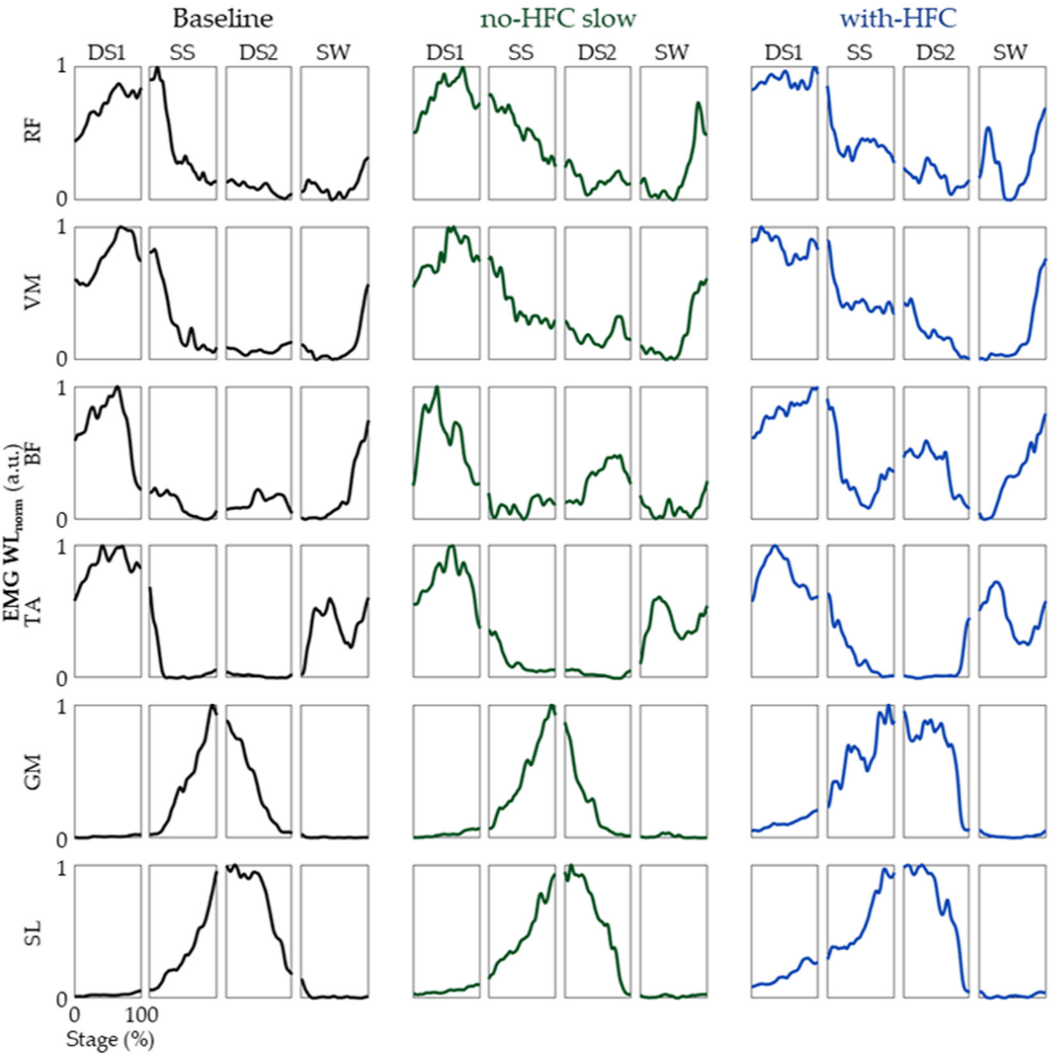

Figure S1. EMG WL$_{norm}$ patterns. The EMG WL$_{norm}$ time traces of the 4 stages are shown for RE, VM, BF, TA, GM, and SL. From left to right, the baseline (black lines), no-HFC slow (green lines), and with-HFC (blue lines) are shown.
